# Supplementary material for: Campylobacter jejuni genotypes are associated with post-infection irritable bowel syndrome in humans
Source: Commun Biol. 2021 Aug 30;4:1015. doi: 10.1038/s42003-021-02554-8 (PMC8405632; doi:10.1038/s42003-021-02554-8)
Supplement: Supplementary file 2 — Supplementary Information [file 42003_2021_2554_MOESM2_ESM.pdf]

Supplementary Figures

Campylobacter jejuni genotypes are associated with post-infection irritable bowel syndrome in humans

Stephanie Peters[1*], Ben Pascoe[2*], Zuowei Wu[3], Sion C. Bayliss[2], Ximin Zeng[1], Adam Edwinson[1], Sakteesh Veerabadhran-Gurunathan[1], Selina Jawahir[4], Jessica K. Calland[2], Evangelos Mourkas[2], Robin Patel[5], Terra Wiens[5], Marijke Decuir[5], David Boxrud[5], Kirk Smith[5], Craig T. Parker[6], Gianrico Farrugia[1], Qijing Zhang[3], Samuel K. Sheppard[2#], Madhusudan Grover[1#]

Supplementary Figure 1: Additional genome details

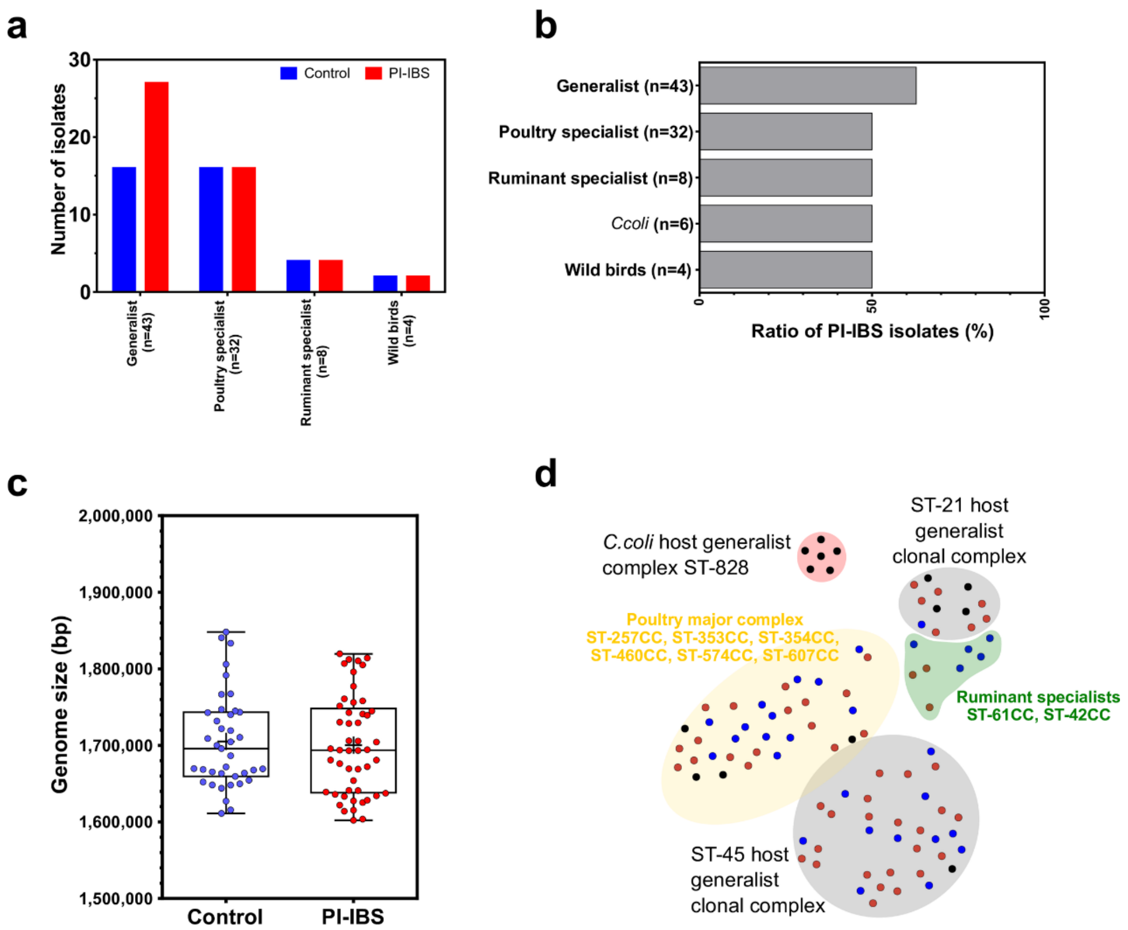

A: Ecological background of PI-IBS and control isolates based on clonal complex association. Generalist isolates were most common, whereas, ruminant and wild birds least common cause of acute enteritis. No significant differences were noted in distribution among the PI-IBS cases and controls. **B:** Number of isolates from each ecological background that led to PI-IBS. Raw data for Figure 1A-B available in Supplementary Data 4. **C:** No differences in genome size between isolates that PI-IBS and control isolates. Box plot shows the 25th and 75th percentile, whiskers extending to the min and max values with all data points shown. Raw data available in Supplementary Data 3. **D:** Isolates cluster according to source ecology based on accessory gene presence (PopPunk). Red dots represent PI-IBS associated isolates, blue dots control isolates and black *C. coli* or where case or control status was unknown.

Supplementary Figure 2: Pangenome visualization

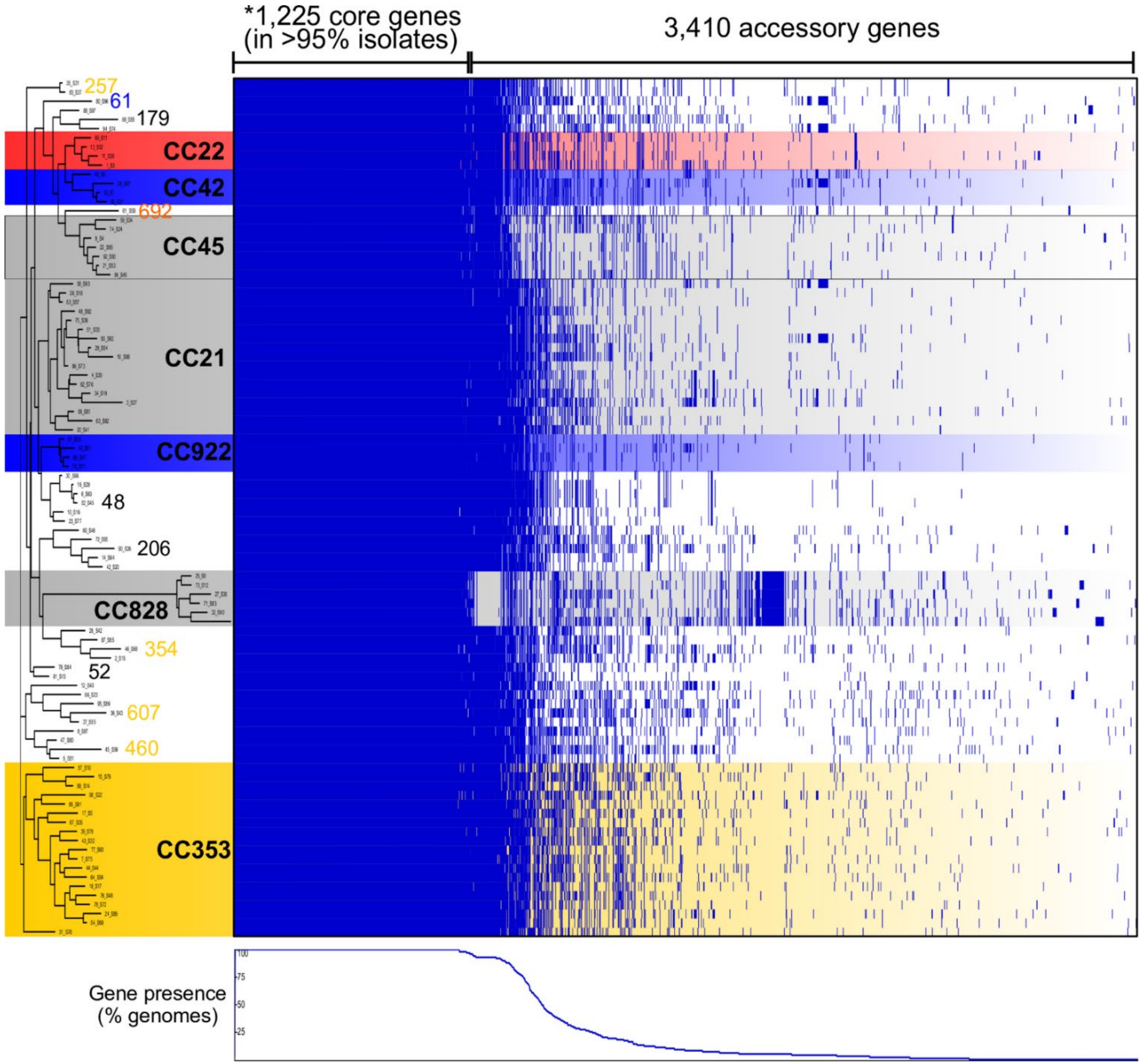

Visualization of the pangenome (PIRATE) with phandango, including estimation of the core (gene present in 95% or more isolates) and accessory genome composition.

Supplementary Figure 3: Additional cytokine responses in colonocytes following exposure to PI-IBS and control C. jejuni isolates

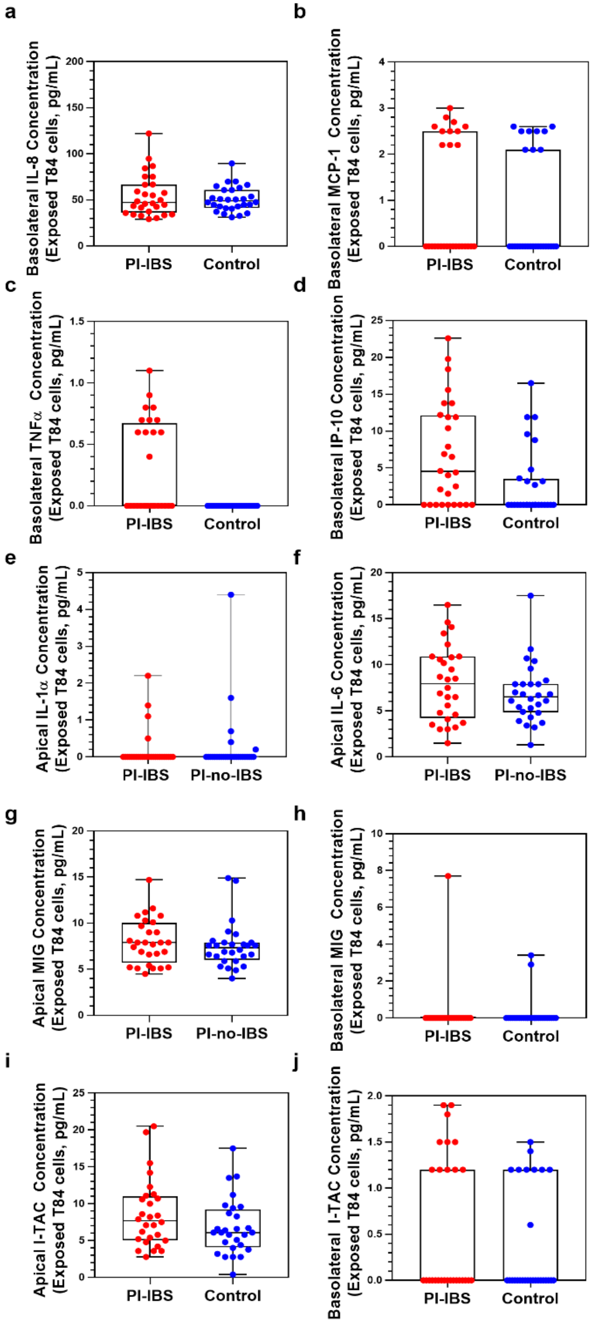

T84 epithelial monolayers were exposed to *C. jejuni* isolates for 24 hrs followed by collection of media from apical and basolateral compartments. **A:** Basolateral IL-8 concentration similar between *C. jejuni* PI-IBS and control isolates. **B:** Basolateral MCP-1 concentration similar between *C. jejuni* PI-IBS and control isolates. **C:** Basolateral TNFα concentration similar between *C. jejuni* PI-IBS and control isolates. **D:** Basolateral IP-10 concentration similar between *C. jejuni* PI-IBS and control isolates. **E:** Apical IL-1α concentration similar between *C. jejuni* PI-IBS and control isolates. **F:** Apical IL-6 concentration similar between *C. jejuni* PI-IBS and control isolates. **G, H:** Apical and basolateral Chemokine (C-X-C motif) ligand 9 or monokine induced by gamma interferon (MIG) concentration similar between *C. jejuni* PI-IBS and control isolates. **I, J:** Apical and basolateral C-X-C motif chemokine 11 (CXCL11) or Interferon-inducible T-cell alpha chemoattractant (I-TAC) concentration similar between *C. jejuni* PI-IBS and control isolates. *All box plots show the 25th and 75th percentile, whiskers extending to the min and max values with all data points shown. Raw data available in Supplementary Data 9
