# Supplementary material for: Campylobacter jejuni genotypes are associated with post-infection irritable bowel syndrome in humans
Source: Commun Biol. 2021 Aug 30;4:1015. doi: 10.1038/s42003-021-02554-8 (PMC8405632; doi:10.1038/s42003-021-02554-8)
Supplement: Supplementary file 3 — Description of Additional Supplementary Files [file 42003_2021_2554_MOESM3_ESM.pdf]

Description of Additional Supplementary Files

File name: Supplementary Data 1.

Description:

File name: Supplementary Data 2.

Description: Alignment (IQ-TREE).

File name: Supplementary Data 3.

Description: Genome details and accession numbers.

File name: Supplementary Data 4.

Description: Isolate typing and presence of accessory genome virulence, antimicrobial resistance genes, Clonal complex, LOS class type and CPS/Penner type.

File name: Supplementary Data 5.

Description: PIRATE pangenome file.

File name: Supplementary Data 6.

Description: Summary of GWAS associated elements.

File name: Supplementary Data 7.

Description: Alignment (CF-ML).

File name: Supplementary Data 8.

Description: Per isolate risk score and risk-score for unique Clonal complexes, LOS class type and CPS/Penner type.

File name: Supplementary Data 9.

Description: In vitro pathogenicity-associated phenotype data.

File name: Supplementary Data 10.

Description: Genome-wide variation in NCTC11168 genes during phenotype experiments.
