# Supplementary material for: Campylobacter jejuni genotypes are associated with post-infection irritable bowel syndrome in humans
Source: Commun Biol. 2021 Aug 30;4:1015. doi: 10.1038/s42003-021-02554-8 (PMC8405632; doi:10.1038/s42003-021-02554-8)
Supplement: Supplementary file 14 — Reporting summary [file 42003_2021_2554_MOESM14_ESM.pdf]

nature research

Corresponding author(s): Madhusudan Grover

Last updated by author(s): Jul 27, 2021

Reporting Summary

Nature Research wishes to improve the reproducibility of the work that we publish. This form provides structure for consistency and transparency in reporting. For further information on Nature Research policies, see our Editorial Policies and the Editorial Policy Checklist.

Statistics

For all statistical analyses, confirm that the following items are present in the figure legend, table legend, main text, or Methods section.

n/a	Confirmed	
☐	☒	The exact sample size (*n*) for each experimental group/condition, given as a discrete number and unit of measurement
☐	☒	A statement on whether measurements were taken from distinct samples or whether the same sample was measured repeatedly
☐	☒	The statistical test(s) used AND whether they are one- or two-sided<br>*Only common tests should be described solely by name; describe more complex techniques in the Methods section.*
☐	☒	A description of all covariates tested
☐	☒	A description of any assumptions or corrections, such as tests of normality and adjustment for multiple comparisons
☐	☒	A full description of the statistical parameters including central tendency (e.g. means) or other basic estimates (e.g. regression coefficient) AND variation (e.g. standard deviation) or associated estimates of uncertainty (e.g. confidence intervals)
☐	☒	For null hypothesis testing, the test statistic (e.g. *F*, *t*, *r*) with confidence intervals, effect sizes, degrees of freedom and *P* value noted<br>*Give P values as exact values whenever suitable.*
☒	☐	For Bayesian analysis, information on the choice of priors and Markov chain Monte Carlo settings
☐	☒	For hierarchical and complex designs, identification of the appropriate level for tests and full reporting of outcomes
☐	☒	Estimates of effect sizes (e.g. Cohen's *d*, Pearson's *r*), indicating how they were calculated

Our web collection on statistics for biologists contains articles on many of the points above.

Software and code

Policy information about availability of computer code

Data collection	Scripts for manipulation of additional PIRATE outputs and treeWAS helper scripts are available on GitHub: https://github.com/SionBayliss.
Data analysis	*Provide a description of all commercial, open source and custom code used to analyse the data in this study, specifying the version used OR state that no software was used.*

For manuscripts utilizing custom algorithms or software that are central to the research but not yet described in published literature, software must be made available to editors and reviewers. We strongly encourage code deposition in a community repository (e.g. GitHub). See the Nature Research guidelines for submitting code & software for further information.

Data

Policy information about availability of data

All manuscripts must include a data availability statement. This statement should provide the following information, where applicable:

- Accession codes, unique identifiers, or web links for publicly available datasets
- A list of figures that have associated raw data
- A description of any restrictions on data availability

Short read sequencing data are available on the NCBI SRA, associated with BioProject PRJNA675124 (http://www.ncbi.nlm.nih.gov/bioproject/675124). Assembled genomes, GWAS summary files and supplementary material are available from FigShare: doi:10.6084/m9.figshare.12493106. Phylogenetic trees can be visualized and manipulated on Microreact for the whole dataset: https://microreact.org/project/CampyIBS and the recombination-free phylogeny used in the GWAS at https://microreact.org/project/CampyIBS-CF.

Field-specific reporting

Please select the one below that is the best fit for your research. If you are not sure, read the appropriate sections before making your selection.

☒ Life sciences ☐ Behavioural & social sciences ☐ Ecological, evolutionary & environmental sciences

For a reference copy of the document with all sections, see nature.com/documents/nr-reporting-summary-flat.pdf

Life sciences study design

All studies must disclose on these points even when the disclosure is negative.

Sample size	For bacterial GWAS, no a priori sample size determination was done considering lack of any prior literature assessing effects of genetic composition on PI-IBS development. Considering the known invasion differences between positive (81-176)and negative (11168) control strains, for in vitro studies, sample size of 28/group (PI-IBS and control) was sufficiently powered to determine differences between high and low virulence strains.
Data exclusions	Only isolates were the case or control status was not known at follow up were excluded from analysis comparing PI-IBS and controls. C. coli isolates were excluded from GWAS analysis considering they are genetically diverse from C. jejuni.
Replication	In vitro studies of C. jejuni adhesion and invasion were performed in triplicate and each triplicate on three different days, for a total of nine observations per strain. For effects on barrier function in CellZScope, each strain was studied in triplicate.
Randomization	NA
Blinding	Personnel performing the in vitro studies of bacterial virulence were blinded to the PI-IBS or control status of the isolates. The case and control status was known to the bioinformatics team as it was essential to determine genomic differences.

Reporting for specific materials, systems and methods

We require information from authors about some types of materials, experimental systems and methods used in many studies. Here, indicate whether each material, system or method listed is relevant to your study. If you are not sure if a list item applies to your research, read the appropriate section before selecting a response.

Materials & experimental systems

n/a	Involved in the study	
☒	☐	Antibodies
☐	☒	Eukaryotic cell lines
☒	☐	Palaeontology and archaeology
☒	☐	Animals and other organisms
☐	☒	Human research participants
☒	☐	Clinical data
☐	☒	Dual use research of concern

Methods

n/a	Involved in the study	
☒	☐	ChIP-seq
☒	☐	Flow cytometry
☒	☐	MRI-based neuroimaging

Eukaryotic cell lines

Policy information about cell lines

Cell line source(s)	T84 human colon cancer cells
Authentication	Authenticated cell line was purchased from ATCC
Mycoplasma contamination	Cell lines were tested for Mycoplasma and were found to be not contaminated
Commonly misidentified lines (See ICLAC register)	NA

Human research participants

Policy information about studies involving human research participants

Population characteristics	Adult female and male patients (>18 years) with stool culture positive Campylobacter enteritis were surveyed 6-9 months post infection to determine the presence of PI-IBS using Rome III criteria. Details on survey strategy and clinical metadata collected are published (Berumen et al., 2020; PMID 32711045). Demographic and clinical symptom data during acute campylobacteriosis was collected. Comparisions were made between patients who developed PI-IBS vs those who did not (controls).
Recruitment	Patients were recruited using a survey (mail followed by telephone). Responder bias can result in overestimation of PI-IBS as those with ongoing GI symptoms are more likely to complete the survery. However, the intent of this study is to compare bacterial genomics and virulence between PI-IBS cases and controls. Hence, it is unlikely that responder bias will have a major influence. Recall bias is also possible in any study inquiring about chronic symptoms. However, this would be expected to be equally distributed among the cases and controls
Ethics oversight	Mayo Clinic and Minnesota Department of Health

Note that full information on the approval of the study protocol must also be provided in the manuscript.

Dual use research of concern

Policy information about dual use research of concern

Hazards

Could the accidental, deliberate or reckless misuse of agents or technologies generated in the work, or the application of information presented in the manuscript, pose a threat to:

No	Yes	
☒	☐	Public health
☒	☐	National security
☒	☐	Crops and/or livestock
☒	☐	Ecosystems
☒	☐	Any other significant area

Experiments of concern

Does the work involve any of these experiments of concern:

No	Yes	
☒	☐	Demonstrate how to render a vaccine ineffective
☒	☐	Confer resistance to therapeutically useful antibiotics or antiviral agents
☒	☐	Enhance the virulence of a pathogen or render a nonpathogen virulent
☒	☐	Increase transmissibility of a pathogen
☒	☐	Alter the host range of a pathogen
☒	☐	Enable evasion of diagnostic/detection modalities
☒	☐	Enable the weaponization of a biological agent or toxin
☒	☐	Any other potentially harmful combination of experiments and agents
